# Association between the triglyceride glucose index and atherosclerotic cardiovascular disease in the general population: analysis of the national health and nutrition examination survey 1999–2004

**DOI:** 10.3389/fendo.2024.1376357

**Published:** 2024-05-21

**Authors:** Sun Jihong, Chen Xiaojie, Lu He, Zhao Yifan

**Affiliations:** ^1^Department of cardiology, the First Affiliated Hospital of Zhengzhou University, Zhengzhou, Henan, China; ^2^Department of Radiology, The People’s Hospital of Jiawang District of Xuzhou, Xuzhou, Jiangsu, China

**Keywords:** triglyceride-glucose index, atherosclerotic cardiovascular disease, coronary artery disease, ischemic stroke, peripheral arterial disease

## Abstract

**Objective:**

The triglyceride-glucose (TyG) index, a reliable substitute indicator of insulin resistance (IR), is considered an independent risk factor for long-term outcomes in patients with cardiovascular disease. However, studies investigating the association between TyG and atherosclerotic cardiovascular disease (ASCVD) are limited and lack direct evidence. We aim to examine the relationship between the TyG index and ASCVD through a comprehensive cross-sectional study.

**Methods:**

Overall, 7212 participants from the 1999–2004 National Health and Nutrition Examination Survey were included. The baseline TyG index was calculated as ln [fasting triglyceride (mg/dL) × fasting blood glucose (mg/dL)/2]. Restricted cubic spline (RCS) regression, univariate logistic regression, and multivariate logistic regression analysis were used to evaluate the association between the TyG index and ASCVD.

**Results:**

In the overall population, a multivariate logistic regression analysis showed that the TyG level was not only positively associated with ASCVD [OR (95%CI): 1.29 (1.01,1.64), *P*=0.042], coronary artery disease (CAD) [OR (95%CI): 1.82(1.33,2.48), *P*<0.001], and stroke [OR (95%CI): 2.68(1.54,4.69), *P*=0.002], but also linearly correlated with all three (*P*-overall<0.001; *P*-non-linear >0.05). Although the TyG index was not associated with peripheral arterial disease (PAD) [OR (95%CI): 1.00 (0.73,1.36), *P*>0.900], it showed a U-shaped correlation with PAD (*P*-overall <0.001; *P*-non-linear= 0.0085), and the risk of PAD was minimized when TyG=8.67. By incorporating the TyG index into the baseline risk model, the accuracy of ASCVD prediction was improved [AUC: baseline risk model, 0.7183 vs. baseline risk model + TyG index, 0.7203, *P* for comparison=0.034]. The results of the subgroup analysis were consistent with those of the main analysis.

**Conclusion:**

The TyG index was independently associated with ASCVD, CAD, and stroke, suggesting that it may serve as a valid indicator for predicting ASCVD in the entire population.

## Introduction

Atherosclerotic cardiovascular disease (ASCVD) is a general term for a group of diseases caused by atherosclerosis that involve blood vessels and the heart throughout the body (1–4), including coronary artery disease (CAD), ischemic stroke, and peripheral arterial disease (PAD), which is the leading cause of death (5–8). It is well known that both triglyceride (TG) and fasting blood glucose (FBG) abnormalities are prone to ASCVD. However, FBG and TG as single indicators are limited in predicting the occurrence of ASCVD, and invasive tests, such as angiography are time-consuming and costly. If a simple and inexpensive clinical indicator can be developed for the early identification of ASCVD risk groups, it will be of great clinical value for the prevention and treatment of this disease.

Insulin resistance (IR) contributes to CAD and plays a key role in the development of type 2 diabetes and atherosclerotic disease (9–11). The hyperinsulinemic hyperglycemic clamp is considered to be the gold standard for quantifying insulin sensitivity. However, it is not commonly used in clinical practice because it is not only expensive, but also time-consuming and labor-intensive to measure. Thus, the homeostasis model assessment of insulin resistance (HOMA-IR) has been proposed as an alternative. Notably, the clinical utility of HOMA-IR is limited owing to the limitations of the insulin measurement methods and their susceptibility to confounding factors. Patients with IR tend to have disturbed glucose and lipid metabolism, which induces a series of inflammatory responses and oxidative stress (12, 13). Based on this theoretical background, the triglyceride-glucose (TyG) index calculated using TG and FBG was proposed as a convenient IR marker in 2008 (14). Similar to other IR markers, the TyG index has been shown to be associated with the risk of several chronic diseases related to diabetes, cardiovascular disease, stroke, and renal microvascular injury (15–20). However, there is limited research on the relationship between the TyG index and ASCVD, particularly due to the lack of validation in large-scale studies based on the general population. To address this knowledge gap, this study selected 7212 adults in the United States who participated in the National Health and Nutrition Examination Survey (NHANES) survey from 1999–2004 to explore the relationship between the TyG index and ASCVD by a cross-sectional study.

## Materials and methods

### Study population

The data for this cross-sectional study were obtained from the NHANES database, a large general population-based cross-sectional survey led by the National Center for Health Statistics (NCHS) that collected information on the health and nutrition of household populations in the United States. This national survey used a complex, multistage, stratified sampling design to identify participants, and oversampled certain populations to ensure a representative sample. This survey has been released every two years since 1999; additionally, all data were validated by the National Center for Health Statistics before being made public and available to the entire population. The NHANES survey was approved by the National Health and NCHS Research Ethics Review Board, and informed consent was obtained from all participants. The NHANES survey data for each period and detailed survey operation manuals, consent forms, and brochures are publicly available on the NHANES website (https://www.cdc.gov/nchs/nhanes).

**Figure 1:**
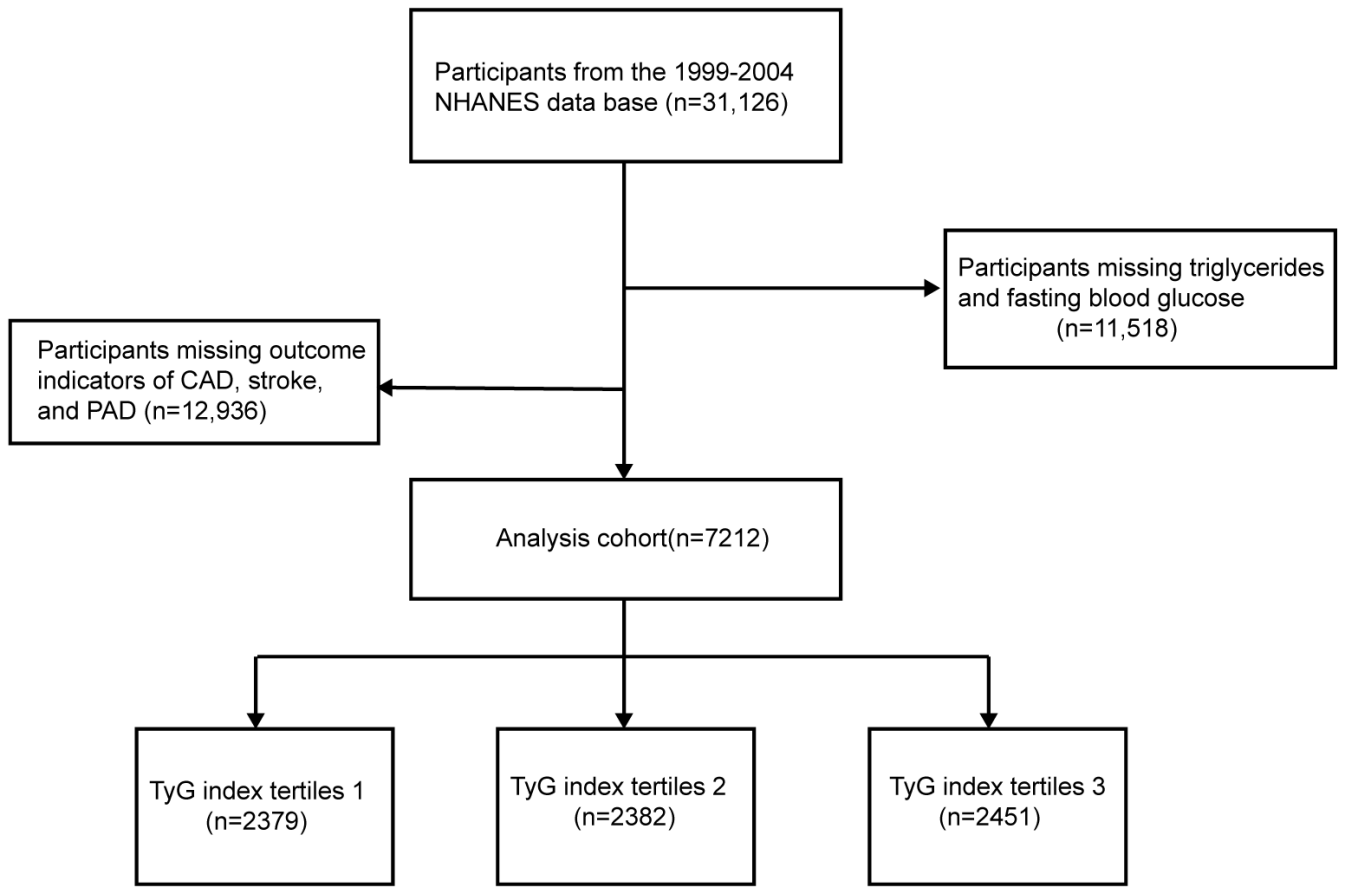
Flow chart for the enrollment of study population.

Adult participants from the 1999–2004 NHANES database were selected to investigate the relationship between the TyG index and ASCVD risk. A total of 31,126 NHANES participants from to 1999–2004 were initially included in this study; 11,518 participants with missing TG and FBG and 12,396 participants with missing outcome indicators of CAD, stroke, or PAD were excluded, and a final total of 7,212 participants were included in this study (**Figure 1**).

### Exposure variable and outcomes

The baseline information of the participants, such as sex, age, ethnicity, household income, education, alcohol and smoking status, and medical history (including hypertension, diabetes, cardiovascular diseases, stroke, and prescription medication use) were obtained from the household interview questionnaire. Physical examination information, such as height, weight, and blood pressure was obtained at mobile screening centers. After resting for at least 5 min, the technician measured the blood pressure in the participant’s right arm using an automated manometer. The systolic blood pressure was measured in both arms (brachial artery) and ankles (posterior tibial artery) using an 8 MHz Doppler probe. The ankle-brachial blood pressure index (ABPI) was calculated as the ratio of the mean systolic pressure of the tibial artery to that of the brachial artery. The participants were requested to fast for more than 8 h before blood samples were drawn to determine the total cholesterol (TC), TG, FBG, high-density lipoprotein cholesterol (HDL-C), low-density lipoprotein cholesterol (LDL-C), creatinine, aspartate aminotransferase (AST), alanine aminotransferase (ALT), and glycosylated hemoglobin A1C (HbA1C) levels, a process that took place at a mobile examination center (MEC). All biological samples were sent to the nearest collaborating laboratory for testing, and all physical examinations and laboratory tests were performed in accordance with the relevant guidelines.

ASCVD was defined as CAD, stroke, or PAD in arterial disease. The diagnosis of CAD and stroke was based on the patients’ previous diagnoses or current symptoms due to data constraints from the NHANES survey. CAD was defined as a previous diagnosis of coronary heart disease, angina pectoris, or a heart attack. Stroke was defined as having been diagnosed by a doctor or professional health advisor that had a stroke. As no questionnaires specifically related to the PAD diagnosis and symptoms were available, we relied on the ABPI as a measurement to define PAD. ABPI provides an effective means of assessing the stenosis between the aorta and ankle by measuring the ratio of ankle artery pressure to the central blood pressure. It exhibits a higher sensitivity than symptomatic diagnosis and has been widely adopted (21, 22). In line with the consensus document by the American Diabetes Association, we defined ABPI<0.9 as indicative of Peripheral Arterial Disease (PAD) (23). The primary outcome was ASCVD and the secondary outcomes were CAD, stroke, and PAD. Notably, the following were used for analyses: 1) HOMA-IR = fasting blood glucose (FBG, mmol/L) × fasting insulin (μU/mL)/22.5; 2) TyG index= ln [fasting TG (mg/dL) × FBG (mg/dL)/2]. Diabetes was defined as follows: 1. Previous diagnosis of diabetes or current use of oral hypoglycemic medication/insulin; 2. Fasting blood glucose level >7 mmol/L; and 3. HbA1c>6.5%. Hypertension was defined as a history of hypertension, current antihypertensive medication use, or maximum systolic blood pressure greater than 140 mmHg/maximum diastolic blood pressure greater than 90 mmHg. Alcohol consumption was defined as a frequency of alcohol consumption >=1 drink/month in the past 12 months.

### Statistical analysis

Statistical analyses were performed using R 4.3.0, and a *P*-value <0.05 was considered statistically significant. While performing the data analyses, we considered sample weights to correct for differential selection probabilities, compensate for possible deficiencies in the eligible population, and adjust for non-coverage and non-response. The missing covariates were supplemented using random forest-based multiple interpolation. TyG was categorized as T1, T2, or T3 based on the tertiles. Quantitative information was expressed using mean ± standard deviation (SD), and qualitative information was expressed as a percentage. Differences in the baseline information between groups with different TyG indices were explored using a variance analysis or the Kruskal–Wallis test for continuous variables and the chi-square test for categorical variables. In addition, a restricted cubic spline (RCS) curve was constructed to evaluate the linear or nonlinear relationships between the TyG index and ASCVD, CAD, stroke, and PAD. The associations between TyG levels and ASCVD, CAD, stroke, and PAD were estimated using multifactorial logistic regression models with 95% confidence intervals (CI). The confounding variables were selected based on the significant differences between the TyG index and risk factors significantly associated with ASCVD. Model 1 is unadjusted. Model 2 was adjusted for age, sex, race, education, and household poverty index (PIR) based on Model 1. Model 3 was adjusted for smoking, alcohol consumption, hypertension, diabetes, HDL-C, LDL-C, FBG, HbA1c, TG, TC, and insulin levels based on Model 2. To evaluate the improvement of the TyG index compared to HOMA-IR, FBG, and TG in traditional ASCVD prediction models, we combined the baseline risk model with each of them to create four new models, analyzed the diagnostic value using receiver operating characteristic curves (ROCs), and calculated the area under the curve to quantify the predictive effect of the four models. The Net Reclassification Improvement (NRI) and Integrated Discriminant Improvement (IDI) were also tested to explore the predictive efficacies of HOMA-IR, FBG, and TG for ASCVD. In addition, we performed sensitivity analyses for specific subgroups, such as sex, age, hypertension, diabetes, and BMI, and used *P* interaction tests to determine whether the confounders interacted with exposure factors.

## Results

### Characteristics of the population stratified by the triglyceride glucose index

The baseline characteristics of the study population are presented according to the TyG tertiles in **Table 1**. A total of 7212 participants were enrolled in this study, of which 3691 (51.2%) participants were male, with a mean age of 60.26 years. Notably, 1841 (25.5%) participants were eligible for the diagnosis of ASCVD, 840 (11.6%) participants had CAD, 300 (4.2%) participants had stroke, and 1054 (14.6%) participants had PAD. The ranges of TyG index for T1-T3 were <8.41, 8.41–8.93, and >8.93, respectively. Individuals with higher TyG levels included more non-Hispanic whites, more males, were older on average, and had a higher prevalence of a variety of disorders, such as hypertension, diabetes, CAD, stroke, PAD, and ASCVD. In addition, a higher TyG index was associated with higher blood indices including FBG, TG, HOMA-IR, ALT, HbA1c, LDL-C, TC, and other blood markers. **Table 2** demonstrated the baseline characteristics of the population grouped according to ASCVD, and the results showed that ASCVD was more common in people who were Non-Hispanic White, older, male, less educated, poorer, smokers, alcohol drinkers, hypertensive, and diabetic. Moreover, patients with ASCVD tended to have higher blood biochemicals, such as TyG, FBG, TG, and HbA1C.

**Table 1:** The characteristics of participants according to TyG index.

Variable	Overall	T1	T2	T3	*P* value
	(n=7212)	(n=2379)	(n=2382)	(n=2451)	
Male (%)	3691 (51.2)	1086 (45.6)	1207(50.7)	1398 (57.0)	<0.001
Race (%)					<0.001
Mexican American	1530 (21.2)	341 (14.3)	528 (22.2)	661 (27.0)	
Other Hispanic	275 (3.8)	68 (2.9)	104 (4.4)	103 (4.2)	
Non-Hispanic White	3959 (54.9)	1313 (55.2)	1322 (55.5)	1324 (54.0)	
Non-Hispanic Black	1238 (17.2)	593 (24.9)	356 (14.9)	289 (11.8)	
Other/multiracial	210 (2.9)	64 (2.7)	72 (3.0)	74 (3.0)	
Age (years)	60.26(13.08)	58.30 (13.53)	61.49 (13.10)	60.96 (12.39)	<0.001
Education (%)					<0.001
Less than high school	2423 (33.6)	680 (28.6)	819 (34.4)	924 (37.7)	
High school graduate or equivalent	1678 (23.3)	540(22.7)	561 (23.6)	577 (23.5)	
Some college or above	3111 (43.1)	1159 (48.7)	1002 (42.1)	950 (38.8)	
PIR	2.78 (1.61)	2.98 (1.64)	2.76 (1.59)	2.60 (1.59)	<0.001
BMI (kg/m2)	28.43 (5.62)	26.71 (5.37)	28.83 (5.85)	29.72(5.18)	<0.001
HbA1c	5.78 (1.13)	5.41 (0.52)	5.59 (0.64)	6.31 (1.63)	<0.001
FBG (mg/dl)	102.23(38.16)	89.44 (11.02)	95.58 (16.13)	121.12(57.93)	<0.001
TG (mg/dl)	154.45(145.94)	73.26 (18.05)	125.45(23.77)	261.45(207.72)	<0.001
HDL-C (mg/dl)	52.66 (16.19)	61.49 (16.87)	52.52 (14.43)	44.23 (12.09)	<0.001
LDL-C (mg/dl)	126.47 (37.76)	118.47 (32.13)	129.44(34.33)	131.35(39.02)	<0.001
ALT (u/l)	25.95 (30.72)	24.22 (45.34)	25.48 (20.21)	28.07 (19.45)	<0.001
AST (u/l)	25.94 (25.47)	26.07 (36.95)	25.80 (19.87)	25.94 (14.17)	0.934
TC (mg/dl)	207.55 (41.05)	195.29 (35.91)	206.97(37.15)	220.02 (45.44)	<0.001
Insulin (uU/ml)	13.03 (15.79)	9.47 (17.05)	17.42 (16.47)	12.08 (12.35)	<0.001
Smoke (%)					<0.001
Never	3338 (46.3)	1177 (49.5)	1128 (47.4)	1033 (42.1)	
Past	1397 (19.4)	463 (19.5)	408 (17.1)	526 (21.5)	
Current	2477 (34.3)	739 (31.1)	846 (35.5)	892 (36.4)	
Drinking (%)	4828 (66.9)	1648 (69.3)	1573 (66.0)	1607 (65.6)	0.012
HOMA-IR	3.61 (5.86)	2.15 (4.70)	2.92 (3.28)	5.71 (7.88)	<0.001
Diabetes (%)	1266 (17.6)	139 (5.8)	296 (12.4)	831 (33.9)	<0.001
Hypertension (%)	4295 (59.6)	1232 (51.8)	1467 (61.6)	1596 (65.1)	<0.001
CAD (%)	840 (11.6)	191 (8.0)	283 (11.9)	366 (14.9)	<0.001
Stroke (%)	300 (4.2)	59 (2.5)	116 (4.9)	125 (5.1)	<0.001
PAD (%)	1054 (14.6)	326 (13.7)	333 (14.0)	395 (16.1)	0.034
ASCVD (%)	1841 (25.5)	509 (21.4)	608 (25.5)	724 (29.5)	<0.001

PIR, poverty index; BMI, body mass index; HbA1c, Glycosylated Hemoglobin, Type A1C; FBG, fasting blood glucose; TG, triglycerides; HDL-C, high-density lipoprotein cholesterol; LDL-C, low-density lipoprotein cholesterol; ALT, alanine aminotransferase; AST, aspartate aminotransferase; TC, total cholesterol; HOMA-IR, homeostasis model assessment of insulin resistance; CAD, coronary artery disease; PAD, peripheral arterial disease; ASCVD, atherosclerotic cardiovascular disease; T, tertile.

**Table 2:** The characteristics of participants according to ASCVD.

Variable	Overall	Non-ASCVD	ASCVD	*P* value
	(n=7212)	(n=5371)	(n=1841)	
Male (%)	3691 (51.2)	2623 (48.8)	1068(58.0)	<0.001
Race (%)				<0.001
Mexican American	1530 (21.2)	1186 (22.1)	344 (18.7)	
Other Hispanic	275 (3.8)	231 (4.3)	44 (2.4)	
Non-Hispanic White	3959 (54.9)	2829 (52.7)	1130(61.4)	
Non-Hispanic Black	1238 (17.2)	963 (17.9)	275 (14.9)	
Other/multiracial	210 (2.9)	162 (3.0)	48 (2.6)	
Age (years)	60.26(13.08)	58.08 (12.53)	66.59 (12.56)	<0.001
Education (%)				<0.001
Less than high school	2423 (33.6)	1709 (31.8)	714 (38.8)	
High school graduate or equivalent	1678 (23.3)	1257(23.4)	421 (22.9)	
Some college or above	3111 (43.1)	2405 (44.8)	706 (38.3)	
PIR	2.78 (1.61)	2.86 (1.62)	2.55 (1.58)	<0.001
BMI (kg/m2)	28.43 (5.62)	28.39 (5.63)	28.56 (5.58)	0.250
HbA1c	5.78 (1.13)	5.72 (1.10)	5.93 (1.20)	<0.001
FBG (mg/dl)	102.23(38.16)	100.44 (36.12)	107.45 (43.16)	<0.001
TG (mg/dl)	154.45(145.94)	152.17 (146.79)	161.12(143.24)	0.023
HDL-C (mg/dl)	52.66 (16.19)	53.44 (16.38)	50.38 (15.42)	<0.001
LDL-C (mg/dl)	126.47 (35.76)	128.46 (35.51)	120.66(35.87)	<0.001
ALT (u/l)	25.94 (30.72)	26.64 (34.52)	23.90 (14.7)	<0.001
AST (u/l)	25.94 (25.47)	26.25 (28.81)	25.02 (10.92)	0.074
Cholesterol (mg/dl)	207.55 (41.05)	209.88 (39.91)	200.77(43.52)	<0.001
Insulin (uU/ml)	13.03 (15.79)	12.08 (10.89)	15.80 (24.92)	<0.001
Smoke (%)				<0.001
Never	3338 (46.3)	2600(48.4)	738 (40.1)	
Past	1397 (19.4)	1082 (20.1)	315 (17.1)	
Current	2477 (34.3)	1689(31.4)	788 (42.8)	
Drinking (%)	4828 (66.9)	3637 (67.7)	1191 (64.7)	0.012
Diabetes (%)	1266 (17.6)	798 (14.9)	468 (25.4)	<0.001
Hypertension (%)	4295 (59.6)	2960 (55.1)	1335 (72.5)	<0.001

The abbreviations are annotated as in **Table 1**.

### Trend test between TyG index and primary and secondary outcomes

**Figure 2:**
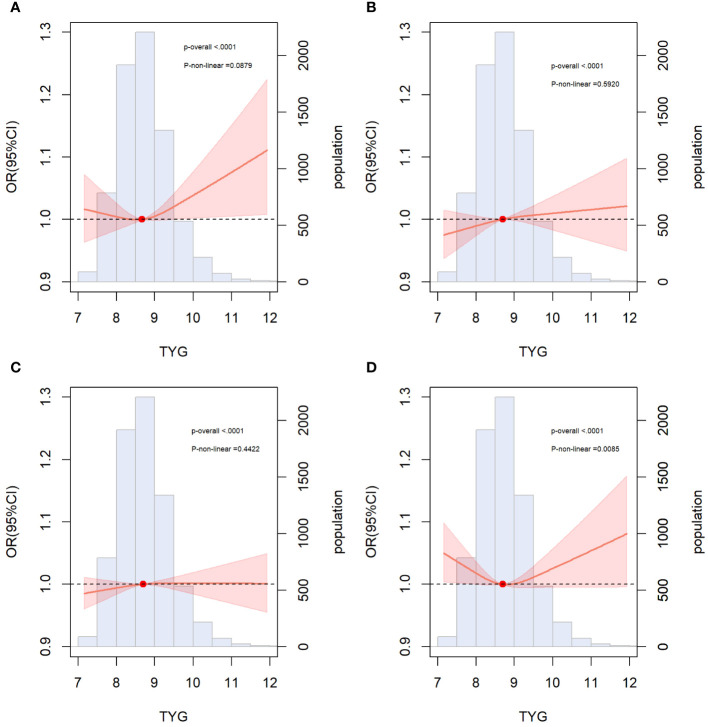
Restricted cubic spline curves for ASCVD, CAD, stroke and PAD by TyG index after covariate adjustment. **(A)** Relationship between TyG index and ASCVD; **(B)** Relationship between TyG index and CAD; **(C)** Relationship between TyG index and stroke **(D)** Relationship between TyG index and PAD. The threshold of statistical significance was set as *P*<0.05. The histograms represented the distribution of people with different TYG index. ASCVD, atherosclerotic cardiovascular disease; CAD, coronary artery disease; PAD, peripheral arterial disease.

RCS regression was used to explore the dose-response relationship between the TyG index and primary and secondary outcomes (**Figure 2**). The results showed that the TyG index was linearly correlated with ASCVD (*P* for nonlinearity=0.088), CAD (*P* for nonlinearity=0.592), and stroke (*P* for nonlinearity=0.442). Therefore, T1 was chosen as the reference when constructing the multivariable logistic regression models for ASCVD, CAD, and stroke. Additionally, the TyG index demonstrated a U-shaped relationship with PAD (*P* for nonlinearity=0.009), with the smallest odds ratio at TyG = 8.67. Therefore, T2 was selected as the reference for developing the PAD model.

### Association of TyG index with ASCVD in overall population

The final confounders included in the model were sex; age; race; education; PIR; FBG, TG, HbA1c, AST, ALT, LDL-C, HDL-C and TC levels; creatinine; hypertension; diabetes mellitus; smoking; and alcohol consumption. The multivariate logistic regression analysis demonstrated an association between the TyG levels and the risk of ASCVD, CAD, stroke, and PAD (**Table 3**). Regarding the risk of ASCVD, the OR and 95% CI for the largest tertile (T3) over the smallest tertile (T1) was [1.75 (1.46,2.11); *P*<0.001) in the unadjusted model 1, [1.47 (1.19,1.81); *P*<0.001) in the partially adjusted model 2, and [1.29 (1.01, 1.64], *P*=0.042) in the fully adjusted model 3. Compared with T1, the risk of CAD in T3 was significantly higher in the unadjusted model 1 [OR (95%CI): 2.52(1.96, 3.25), *P*<0.001], partially adjusted model 2 [OR (95%CI): 2.12 (1.61, 2.79), *P*<0.001], and fully adjusted model 3 [OR (95%CI): 1.82(1.33, 2.48), *P*<0.001] The risk of stroke was significantly increased at the highest TyG level compared to the lowest TyG level in model1[OR (95%CI): 2.71(1.82, 4.03), *P*<0.001], model2 [OR (95%CI): 2.43(1.57, 3.75), *P*<0.001], and model3[OR (95%CI): 2.68(1.54, 4.69), *P*=0.002]. The risk of PAD with different TyG levels was not statistically significant in the all-adjusted model [OR (95%CI): 1.00 (0.73, 1.36), *P*>0.900] (**Table 3**). In addition, individuals with higher TyG levels have a greater risk of ASCVD (*P* for trend<0.001), CAD (*P* for trend<0.001), and stroke (*P* for trend=0.002) (**Table 3**).

**Table 3:** Association of TyG index with the risk of ASCVD, Stroke, CAD, and PAD.

	Case	N	Model1		Model2		Model3	
			OR (95%CI)	*P* value	OR (95%CI)	*P* value	OR (95%CI)	*P* value
ASCVD								
T1	509	2379	Ref		Ref		Ref	
T2	608	2382	1.42 (1.14,1.78)	0.003	1.17 (0.94,1.47)	0.200	1.13 (0.88,1.45)	0.300
T3	724	2451	1.75 (1.46,2.11)	<0.001	1.47 (1.19,1.81)	<0.001	1.29 (1.01,1.64)	0.042
P for trend				<0.001		<0.001		<0.001
CAD								
T1	191	2379	Ref		Ref		Ref	
T2	283	2382	1.59 (1.22,2.08)	0.001	1.27 (0.98,1.65)	0.068	1.23 (0.92,1.65)	0.200
T3	366	2451	2.52 (1.96,3.25)	<0.001	2.12 (1.61,2.79)	<0.001	1.82 (1.33,2.48)	<0.001
P for trend				<0.001		<0.001		<0.001
Stroke								
T1	59	2379	Ref		Ref		Ref	
T2	116	2382	2.43 (1.6,3.70)	<0.001	2.1 (1.39,3.18)	<0.001	2.23 (1.49,3.33)	<0.001
T3	125	2451	2.71 (1.82,4.03)	<0.001	2.43 (1.57,3.75)	<0.001	2.68 (1.54,4.69)	0.002
P for trend				<0.001		<0.001		0.002
PAD								
T1	326	2379	Ref		Ref		Ref	
T2	333	2382	1.19 (0.91,1.54)	0.200	1.02 (0.79,1.33)	0.900	0.99 (0.73,1.35)	>0.900
T3	395	2451	1.27 (1.00,1.62)	0.054	1.09 (0.85,1.40)	0.500	1.00 (0.73,1.36)	>0.900
P for trend				<0.001		<0.001		0.931

OR, odds ratio; CI, Confidence Interval; Other abbreviations are shown in **Table 1**.

### Incremental effect of the TyG index for predicting ASCVD

**Figure 3:**
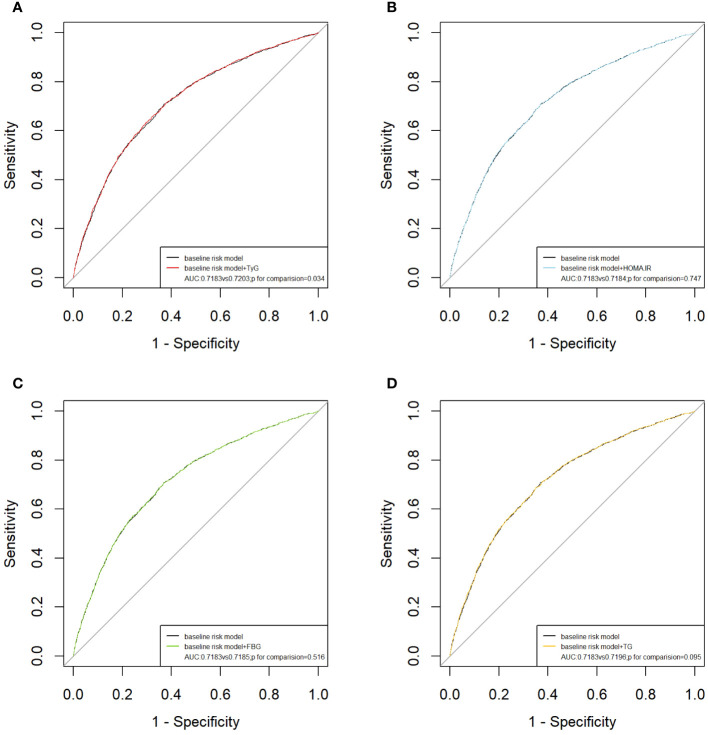
ROC curves for the evaluation of TyG index and other risk factors on the diagnostic performance of ASCVD. **(A)** Baseline risk model vs baseline risk model + TyG index groups; **(B)** Baseline risk model vs baseline risk model + HOMA-IR groups; **(C)** Baseline risk model vs baseline risk model + FBG; **(D)** Baseline risk model vs baseline risk model + TG. Baseline risk models included sex, age, LDL-C, ALT, AST, TC, insulin, hypertension, diabetes, smoking, alcohol consumption. AUC, area under the curve; TyG, triglyceride-glucose; TG, triglyceride; FBG, fasting blood glucose. HOMA-IR, homeostasis model assessment of insulin resistance.

We incorporated traditional cardiovascular risk factors, such as sex, age, race, total cholesterol, LDL-C, hypertension, diabetes, smoking, and alcohol consumption, into the baseline risk model. Subsequently, we individually introduced the TyG index, HOMA-IR, FBG level, and TG level to construct four new models. The predictive performance of these models was evaluated by plotting ROC curves and calculating the AUC. Notably, our analysis revealed that only the inclusion of the TyG index [AUC: baseline risk model, 0.7183 vs. baseline risk model + TyG index, 0.7203, *P*=0.034] significantly improved the base model, with an NRI of 0.009 (*P*=0.110) and an IDI of 0.002 (*P*<0.001) (**Table 4**). However, the addition of HOMA-IR [AUC: baseline risk model, 0.7183 vs. baseline risk model + HOMA-IR index, 0.7184, *P*=0.747], FBG [AUC: baseline risk model, 0.7183 vs. baseline risk model + FBG, 0.7185, *P*=0.516], and TG [AUC: baseline risk model, 0.7183 vs. baseline risk model + TG index, 0.7196, *P*=0.095] did not lead to a statistically significant enhancement in model performance (**Figure 3**).

**Table 4:** Incremental effect of TYG, HOMA.IR, FBG and TG for predicting ASCVD.

	AUC	P for comparisons	NRI (95%CI)	p value	IDI (95%CI)	p value
Baseline risk model	0.7183	Ref	Ref	Ref	Ref	Ref
+TYG	0.7203	0.034	0.009 (-0.002,0.020)	0.110	0.002 (0.001,0.004)	<0.001
+HOMA.IR	0.7184	0.747	0.002 (-0.003,0.007)	0.339	0.001 (-0.001,0.001)	0.156
+FBG	0.7185	0.516	0.001 (-0.001,0.005)	0.792	0.001 (-0.001,0.001)	0.465
+TG	0.7196	0.095	0.001 (-0.008, 0.009)	0.933	0.002 (0.001, 0.003)	<0.001

NRI, Net Reclassification Improvement; IDI, Integrated Discriminant Improvement; AUC, area under the curve. Other abbreviations are listed in **Table 1**.

### Sensitive analysis of association of TyG with ASCVD

**Figure 4:**
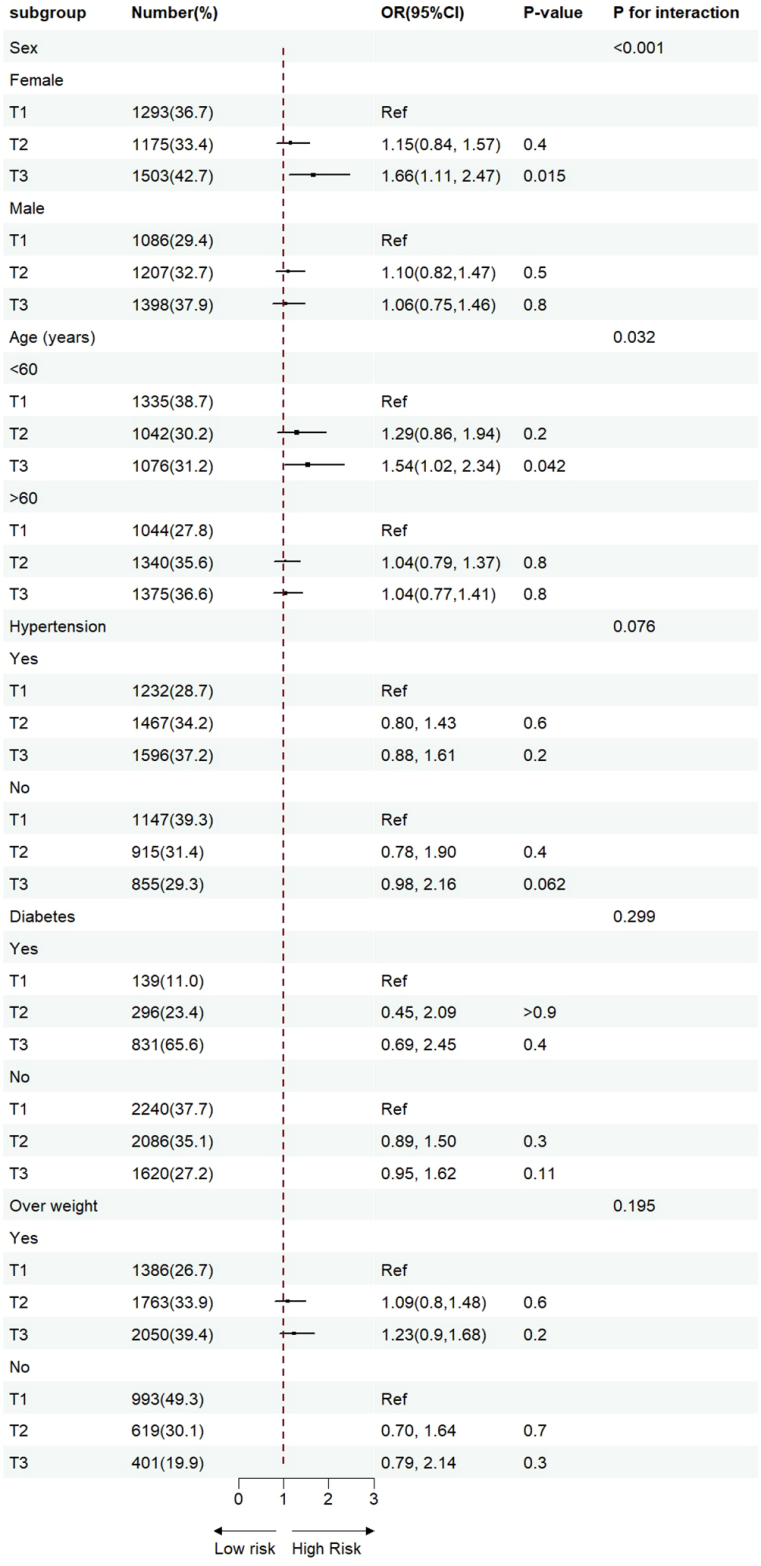
Sensitivity analysis for the impact of TyG index on ASCVD. The red vertical solid line represents OR= 1. The threshold of statistical significance was set as *P*<0.05.

**Figure 5:**
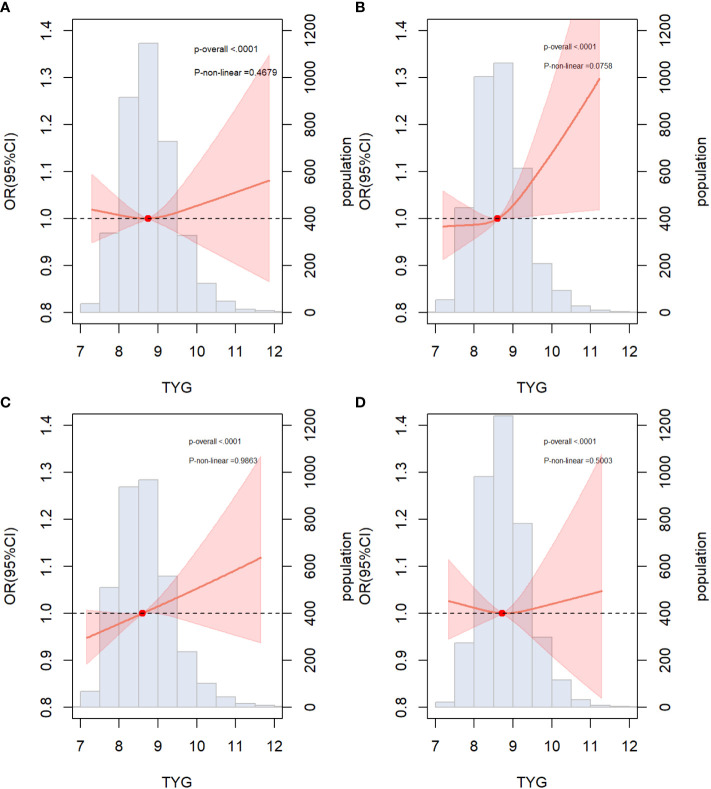
Associations between TYG index and ASCVD in specific subgroups **(A)** Male; **(B)** Female; **(C)** Non-elderly (age<60); **(D)** Elderly (age≥60); The threshold of statistical significance was set as *P*<0.05. The histograms represented the distribution of people with different TYG index levels. ASCVD, atherosclerotic cardiovascular disease.

The results of the subgroup analysis stratified by sex, age, diabetes, hypertension, and BMI are shown in **Figure 4**. Overall, the participants in the highest TyG index tertile had significantly higher odds of developing ASCVD in subgroups. Notably, significant interactions were observed between the TyG index and sex (*P* for interaction <0.001) and age (*P* for interaction = 0.032), and no interaction was found for the other confounding variables. Furthermore, the TyG index was positively associated with the incidence of ASCVD in female individuals [OR (95% CI): 1.66(1.11,2.47) and middle-aged individuals [OR (95% CI): 1.54 (1.02, 2.34]). Building upon these findings, we further explored the relationship between the TyG index and ASCVD in subgroups stratified by sex and age using multivariate-adjusted RCS analysis. As shown in **Figure 5**, while a linear association trend between the TyG index and ASCVD was observed in all subgroups (P for overall <0.001, all P for nonlinear >0.05), a significant positive correlation between the TyG index and ASCVD was evident specifically in the female and middle-aged subgroups.

## Discussion

To the best of our knowledge, this is the first large-scale cross-sectional study to explore the relationship between TyG index and ASCVD. The results showed that The TyG index remained positively associated with the risks of ASCVD, CAD, and stroke after correcting for various confounders. The TyG index was U-shaped and associated with the risk of PAD, with the lowest risk occurring when the TyG index was 8.67. The addition of the TyG index significantly increased the predictive efficacy of the basic model compared with HOMA-IR, FBG, and TG. In summary, the TyG index can serve as a reliable tool for predicting ASCVD risk, thereby optimizing ASCVD prevalence stratification in the general population.

Numerous scholars have focused on the association between the TyG index and the risk of single diseases, such as CAD, stroke, PAD, and atherosclerosis; however little attention has been paid to ASCVD. ASCVD, as a composite outcome, is more closely associated with all-cause mortality and adverse cardiovascular event outcomes than a single disease (24–26), and the early identification and management of patients with high-risk ASCVD is of greater clinical value. Hua et al. simulated the incidence of ASCVD in participants over the next 10 years and found that the TyG index was associated with an increased risk of ASCVD (27). Previous studies have demonstrated the promising potential of the TyG index in predicting the risk of CAD (28, 29) and the prognosis of adverse cardiovascular events in patients with acute coronary syndrome (ACS) (30, 31), stable CAD (32, 33), or non-obstructive CAD (34). A high TyG index is also independently associated with stroke (35). In contrast, Wu et al. found no statistically significant differences in the risk of stroke in those with the highest TyG index compared to those with the lowest TyG index; however, there was a significant linear trend (36). In our study, the TyG index was not only positively correlated with the risk of stroke, but also showed an overall linear trend. From the available studies, it is inconclusive whether the TyG index can be used as an independent predictor of stroke; however, individuals with higher TyG levels tend to have a higher risk of stroke. Evidence of the relationship between the TyG index and PAD is limited. Gao et al. found that in 12,320 non-PAD community participants, a higher TyG index was associated with an increased risk of PAD (37). However, after adjusting for relevant risk factors, we did not find a significant correlation between the TyG index and the risk of PAD, but only observed a U-shaped trend between the TyG index and PAD risk. This difference may be due to our diagnosis of PAD being based on ABPI rather than angiography or symptomatic standards, which could lead to missed diagnoses of mild-to-moderate or early stage PAD, thereby affecting the assessment of the correlation between the TyG index and PAD risk. Second, from a statistical perspective, artificially categorizing the TyG index as a categorical variable in our study may have led to the loss of some information, reducing the sensitivity and accuracy of the analysis, and thereby affecting the discovery of a correlation between the TyG index and risk of PAD. Additionally, the TyG index can represent, to some extent, the nutritional level of participants; both malnutrition and over-nutrition could increase the risk of PAD, which may explain the U-shaped correlation between TyG and PAD (38–40).

Notably, sex and age interacted with the TyG index to predict ASCVD, and the relationship between TyG levels and ASCVD was prominent in the female and middle-aged groups, but not in the male and older groups. Previous studies have repeatedly mentioned the superiority of the TyG index in predicting cardiovascular and atherosclerosis-related diseases in the female population (41, 42), which is generally in line with the results of our study. This difference can be explained by the gender insulin hypothesis (43). Previous studies have found that individuals of female sex tend to be more insulin resistant than male individuals because of the differences in hormone levels, fat distribution, metabolism, genetic and hereditary factors and lifestyle factors (44–46). As the amount of estrogen wanes,IR is observed to increase in women after entering menopause (47), explaining why there are more women with type 2 diabetes. Besides, the interaction between TyG and age has also drawn our attention. After a 7-year follow-up of 2923 patients with cardiovascular disease, Wang et al. found that the TyG levels were a promising marker for predicting the all-cause mortality in middle-aged patients, but not in elderly patients (48). Similarly, middle-aged patients with higher levels of TyG index awere more likely to experience major adverse cardiovascular events (49). However, the exact reasons for this pattern remain to be discussed. The most likely reasons are two-fold. Firstly, younger individuals were more likely to developing IR (50), leading to elevated TyG levels in this population (51, 52). Secondly, the TyG index measurements in the elderly awere susceptible to more confounding factors, such as the presence of various diseases, concomitant poor nutritional status, and altered lipid levels. Therefore, the TyG index might not accurately reflect the IR in older adults compared to younger adults. Overall, the reasons why the female and middle-aged individuals are more prone to IR are likely multifaceted, and further research is needed for a comprehensive understanding of its mechanisms

The mechanism underlying the correlation between the TyG index and ASCVD has not yet been clearly elucidated; however, we believe that this could be explained by the IR doctrine. First, an imbalance in the glucose and lipid metabolism induced by IR leads to inflammation and oxidative stress, resulting in the development of atherosclerosis (53, 54). Second, IR plays a key role in the progression of atherosclerosis by promoting the apoptosis of macrophages, endothelial cells, and vascular smooth muscle cells. In addition, IR with hyperglycemia induces hyperglycosylation, which promotes smooth muscle cell proliferation, collagen cross-linking, and collagen deposition, and is closely associated with a substantial increase in the incidence of vascular fibrosis and stiffness. The cascade of pathological changes associated with IR ultimately leads to an increased incidence of atherosclerosis, cardiovascular diseases, and all-cause mortality.

We also found that after the addition of HOMA-IR, FBG, TG, and TyG grades to the base model, only the TyG index optimized the model, whereas HOMA-IR, FBG, and TG failed to optimize the model, indicating that the TyG index, although calculated from FBG and TG, has a higher diagnostic value, probably because it represents both lipid and glycemic factors and has a higher correlation with IR. HOMA-IR is commonly used for the indirect assessment of IR. However, its clinical utility is limited by the testing methods and costs. Compared to HOMA-IR, the TyG index not only has a higher predictive value for ASCVD, but also does not increase medical expenses, offering significant clinical applicability. However, it has to be mentioned that ASCVD is a dynamic, progressive disease and treatment should be initiated according to the patient’s specific situation, which makes the use of baseline TyG index as a prognostic marker less certain.

This study had several limitations. First, this was an observational study; therefore, it was difficult to exclude the effects of confounding factors. Second, the diagnoses of CAD and stroke are based on self-reported medical history, whereas the diagnosis of PAD is based on ABPI rather than angiography, which may miss patients in the early stages of the disease or those who have not sought medical attention. Third, the data on triglyceride and glucose levels were measured only at baseline and the effect of changes in TyG levels over time on ASCVD was not considered. Furthermore, the use of lipid- and glucose-lowering medications was not considered. Finally, due to the cross-sectional nature of this study, the research results can only indicate an association between the TyG index and high ASCVD risk, but cannot establish a causal relationship between the two. Further longitudinal studies are required to clarify the causal relationship between TyG index and ASCVD.

## Conclusion

In the general population, our study demonstrated that the TyG index was positively associated with the occurrence of ASCVD and had a certain predictive value. However, ASCVD is a dynamic and progressive disease; therefore, enabling the use of the TyG index in real time to dynamically predict the risk of ASCVD is the next challenge for our team.

## Data availability statement

The original contributions presented in the study are included in the article/supplementary material, further inquiries can be directed to the corresponding author/s.

## Ethics statement

The studies involving humans were approved by National Health and Nutrition Examination Survey. The studies were conducted in accordance with the local legislation and institutional requirements. Written informed consent for participation in this study was provided by the participants’ legal guardians/next of kin.

## Author contributions

JS: Data curation, Formal analysis, Methodology, Project administration, Writing – original draft, Writing – review & editing, Conceptualization. XC: Data curation, Formal analysis, Methodology, Project administration, Writing – original draft, Writing – review & editing, Investigation. HL: Data curation, Formal analysis, Investigation, Methodology, Project administration, Writing – original draft, Writing – review & editing. YZ: Data curation, Investigation, Writing – original draft.
